# Tothe-Lore

**Published:** 1899-01

**Authors:** George L. Parmele

**Affiliations:** M.D., D.M.D. (Harv.), Hartford, Conn.


### International Dental Journal.

Vol. XX. | January, 1899. | No. 1.

### Original Communications.[1]

### "TOTHE-LORE." [2]

BY GEORGE L. PARMELE, M.D., D.M.D. (HARV.), HARTFORD, CONN.

> "Aloft in rows large poppy-heads were hung,—
> In this place, drugs, in musty heaps decay'd,
> In that, dry'd bladders and drawn teeth were laid."

At first thought one would hardly imagine that the field to which I invite you this evening could afford sufficient material to allow of the devotion of much time to its consideration.

The call for the meeting states that I will read a paper on "Tothe-Lore," but a title so dignified is hardly appropriate for what I intend to offer; let it pass rather as a compilation of items gathered and noted down from time to time through many years,—items culled from every conceivable source, ranging from ponderous tomes to the old-time family almanac.

From this mass of material I have selected some examples with which to bore you.

Philosophy is said to console a man under disappointment, although Shakespeare asserts that it is no remedy for toothache. Assume, then, the air of a Stoic and lend me your ears. The days of miracles and chivalry, we are told, have passed,—witches, fairies, ghosts, goblins, and devils are laid full many a fathom deep in the

---

[1] The editor and publishers are not responsible for the views of authors of papers published in this department, nor for any claim to novelty, or otherwise, that may be made by them. No papers will be received for this department that have appeared in any other journal published in the country.

[2] Read before the New York Institute of Stomatology, October 4, 1898.

ocean of oblivion, but charms and superstitions still abide with us, and one comes in contact with them almost daily.

How often do we meet those who will not undertake a new work or a journey on Friday, who exclaim when salt is spilled, or throw up their hands in "holy horror" when a mirror is broken!

These remnants of superstition originated in antiquity, and have been fostered by an age of credulity. Myths and superstitions, the wearing of charms and amulets, are common to many lands. Every country has its magic for the relief of pain and cure of disease; belief in it is not confined to the ignorant and uncultured, and no matter how great the distance between the countries a similarity in their myths and traditions will be observed.

Frequently actual prayers, exhortations, and orisons, not always reverential, often a mere form without meaning, are employed.

The constituent elements of folk-lore, items which we gather from day to day, are survivals of a condition of human thought lost in the obscurity of a dim past. Fontenelle, a writer of the last century, shrewdly remarked that "all nations made the astonishing part of their myths while they were savage and retained them from custom and religious conservatism."

"Folk-lore" is a term first suggested by Mr. Thoms, in 1846, to designate "that department of the study of antiquities and archæology which embraces everything relating to ancient observances and customs, to the notions, beliefs, traditions, superstitions, and prejudices of the common people." My title "Tothe-Lore," or "folk-lore of the mouth," is an adaptation of this term to the special line along which I am working.

"Evolution in folk-lore" is a fascinating study, one which I hope to follow, but in which I have as yet hardly passed the stage of a collector.

Remembering Mrs. Glass's advice in cooking, "first catch your rabbit," let us, before dealing with the teeth themselves, observe a few of the caprices, curiosities, and maladies attending their passage through the gum, as well as some of the methods suggested to alleviate all difficulties thereupon attendant.

It is said that the baby who cuts its teeth hard will be successful in every way; on the other hand, the proverb "Soon toothed, soon turfed" signifies that a child which cuts its teeth early will be short lived. This is an English proverb, and the Scotch have as an equivalent "Soon tod, soon God." Some primitive tribes are in fear and dread of children whose upper teeth erupt before the lower and hasten to kill them, as do the Basutos, Wakikuyu, Wanika, Wasawahili, and Wazegua. Among the Wazaramo, another African people, such children are either put to death, given away, or sold to a slave-dealer, for the belief is that, through them, sickness, misfortune, or death will enter the house. The Arabs of Zanzibar, after reading from the Koran, administer to such a child an oath that it will do no harm, making it nod assent with its head.

In the *Popular Science Monthly* I read that "the birth of a child among the Bondei people of Africa is attended, according to the account of the Rev. G. Dale, missionary, by many great perils, for if a single condition regarded as unfavorable occurs, the infant is strangled at once. Its life is in danger again at the time of teething, for it may be so incautious as to let its upper teeth protrude first, and if this is the case it is held unlucky, and will almost certainly be killed. Even if it is allowed to live it will be in perpetual danger, and any disaster that happens to its parents will be attributed to it. If, however, the under teeth protrude first, the child's moral character is established. The boy cannot, however, enter the house in which the unmarried men sleep till he has been publicly welcomed. For this ceremony, all the boys and girls assemble and the father brings the child out to show them that the lower teeth have protruded first. Then every house contributes Indian corn, and the children pound and eat it, after which the boy is regarded as one of them."

In some parts of England it is unlucky for a child to see itself in the mirror before it cuts its teeth, as it will be vain and proud. If a child would be lucky, it must cut its teeth on the mother's marriage ring. Gold, at any rate, should be used to bite upon.

A mole's foot tied by a string and hung around the neck while teething is believed (in and around Washington, D. C.) to aid the process. Perhaps this idea comes from the old doctrine of signatures, as, like the embryo tooth, the mole's foot burrows around in the dark. In some drug-shops, and in the markets in Georgia, the forefeet of moles are found for sale as aids in the eruption of the teeth of colored children.

It is said by old women that teething is made much easier by placing around the child's neck a string of "Job's tears," which consist of the dried berries of *croix lacryma*,[1] which are supposed to have a great soothing power.

---

[1] Croix lacryma is a grass native to the East Indies and Japan. The large, round, shining fruit has, when young, some resemblance to heavy drops of tears, hence the fanciful specific name. The medicinal effects are said to be tonic and diuretic.

"The well-known toy," says Brand, "and a piece of coral at the end, which is generally suspended from the necks of infants to assist them in cutting their teeth, is supposed to have originated in an ancient superstition, which regarded coral as an amulet against fascination. It was thought, too, to preserve and fasten the teeth in man." Plat, in his "Jewel Home of Nature and Art," says, "Coral is good to be hanged about children's necks, as well as to rub their gums, as to preserve them from the falling sickness."

The following I copied from a quaint old medical work, entitled, "An Essay of the Pathology of the Brain and Nervous Stock, in which Convulsive Diseases are Treated of," by Thomas Willis, of Christ Church, Oxford. Translated out of the Latin, London, 1681.

For the "convulsions of teeth-breeding . . . bleeding and the seton are recommended, then the following powder should be given in a spoonful of Jalap, for three days, morning and evening. Take of Human Skull prepared, of the root of male Pæonie, each, ʒi; of Powder of Pearls, ʒss; White Sugar, ʒi. Mingle them and make a very fine powder." From an unrecorded source I ascertained that for a child to cut its first tooth in the upper jaw is regarded in the South as a sign that the child will die in infancy. This idea was probably an African importation.

In North Carolina, when a nurse is so inconsiderate as to hold a baby out of an open window, or to allow it to see its own image in a mirror, the negroes believe that difficult teething is produced, the charm to cure which is a necklace of alligator's teeth, or to rub the gums with the ear of a rabbit.

That the time of the appearance of the teeth was often irregular was known to Shakespeare, for in Richard III., act ii., sc. iv. he makes York say, "Marry, they say, my uncle grew so fast that he could gnaw a crust at two hours old; 'twas full two years ere I could get a tooth. Grandam, this would have been a biting jest."

In a "Commonplace Book," written by one Thomas Rawlins, of Pophills, between the years of 1724 and 1734, occur the following entries. (For these and many such I am indebted to *Notes and Queries.*) "There lives in Mill street, in Belfast, in Ireland, 1731, one Jane Hooks, of one hundred and twelve years of age, who has her memory and apetite as well as when she was but twenty years old, and she has got a new sett of teeth wch has drove out all ye old stumps."

"Robt. Lyon, of ye city of Glasgow, aged one hundred and nine years, who was in service of Charles I. and who has got a new sett of teeth recovered his sight in a wonderful manner."

" Mrs. Page at ye Royal Oak, in Barnaly Street, Southwark, aged ninety years and upwards, has lately cut six great teeth in ye upper jaw, in June, 1732. . . . Had not a tooth in her head these twenty years past."

"Margaret White, of Kirkaldy in Scotland, aged eighty seven, who has been toothless for many years, has just got eight new and fresh teeth, April, 1732."

This from proceedings of the Suffolk Institute of Archæology: "Dying in 1669 she [the widow of John Croftes] bequeathed it [the Hall] to the Hon. Edward Progers of London. 'The gay Progers' who, according to Le Neve, died on the thirty first of December or the first of January, 1713, aged *ninety six* of the anguish of cutting teeth; he having cut four new teeth and had several ready to cut wh. so inflamed the gums that he died thereof."

H. M., in *Notes and Queries*, relates the case of a patient, aged seventy-five, who was laboring under a singular form of mental derangement. Among other points he notes (June, 1843): "A remarkable circumstance in this case is that she has cut an incisive tooth in the lower jaw, and is now cutting another, which fact confirms her in the strange belief that she is leading a *post mortem* existence, and has commenced at infancy again; for upon one of her daughters asking me if I thought it probable she would die, she exclaimed angrily, 'How can I die twice? I am only a child; see, I have not cut all my teeth yet.'"

Another correspondent in the same journal writes: "So far from being an extraordinary case, it really is a most common event, and I will venture to assert that there are very few persons who arrive at my age who have not had three sets of teeth. I can speak from experience. First I had my infantine set; next I had the set which, after serving me usefully for many years, gradually decayed and left me, and now I have a third set from which, I can truly say, *I suffered much cost* in the *cutting* by an eminent dentist in the West end—Septuagenarius et plus."

Bacon, in his "Natural History," speaking of the Countess of Desmond, who lived in the "Reigne of King Edward IV.," of whom it is asserted that she lived one hundred and forty years, says, "She did *dentire* [produce teeth] twice or thrice, casting her old teeth and others coming in their place."

To relieve the monotony, the following may be in order:

"A group of matrons, seated on the piazza of a popular summer resort, were discussing the pearly teeth of a well-known actress, and branched off to criticising the molars and bicuspids of their friends. 'Will you believe it,' remarked one well-preserved personage with a hyphenated name, displaying an admirable development of some width and whiteness, 'that my wisdom-teeth have not yet grown?' A second of dead silence ensued. It was broken by a male voice from the outer edge of the circle,—'Some century-plants never bloom.' The identity of the commentator remains undisclosed."

Turning now to the other extreme, we read that "Marcus Curius, nicknamed *Dentatus*," had all his teeth at birth; Richard III. did the same; and Jacobi reported the case of a Spanish dwarf who was born with all his teeth; and many more such cases could be given. "In the register of burials at Gayton-le-Marsh, Lincolnshire, duly certified to by the curate, is the following: 'Elizabeth Cook, a poor woman aged eighty six, who never had a tooth, was buried January 11, 1798.'"

Since writing this paper Mr. Bates, librarian of the Connecticut Historical Society, handed me the following from the "Simsbury Records:" "Sarah Slater first Daughter of Elias and Sara Slater was born febuary the Sixth day 1716/17 which was wensday—11 aclock at night and baptised the 10th day—the 16 day thare apeared an uper fore toot the 19th day at one of the clok after noon it came quit out."

Let us close the section relating to dentition by guessing the following charade, from a little book entitled "A Century of Charades:"

"My first pours out at early teas;
My second's anything you please;
My whole's the cause of much disease."[1]

Having safely conducted these useful organs on their journey from the "primitive groove" to the light of day, let us see how they have been christened and what names have been given them.

Leaving to the philologist the task of dealing with their various names and their derivations among the many races of the earth from early time, we will direct our attention to some of the more strictly folk-names.

---

[1] Answer, teething (tea thing).

Thus we have "Jenny wi' the Airn teeth," a Scotch painted devil, bogie, or imaginary being with iron teeth, employed to frighten little children into obeying.

> "'Tis the eye of childhood
> That fears a painted devil."
> —SHAKESPEARE.

Frederick II., Elector of Brandenburg (1657–1713), was nicknamed Irontooth (Dent de Fer). A patient furnished me with the quotation, "Put your green teeth into that," referring, no doubt, to that unsightly, chlorophyllaceous stain often seen on the face of incisors. Another patient informs me that in North Carolina protruding incisors are called "butter-teeth." "Buck-teeth" has also been used in speaking of this same deformity. Dents barrés, or barred teeth, are the molar teeth when the roots are spread or tortuous, so that they cannot be extracted without being broken, or without a portion of alveolus being removed. Some old-time names for teeth are fang-tooth, the eye-tooth, wang-tooth, a molar, lag-teeth, wall-teeth, azzle-teeth. Axle-teeth and cheek-teeth are also synonymes for molar-teeth. Then we have wit-tooth, wisdom-tooth, or dens sapientiæ. The canine has been called eye-tooth, dog-tooth, and pug-tooth, a Devonshire word. The molar has been mentioned as pugging tooth, comparing it to a machine called a "pugging mill," by which clay is worked to blend its materials and render it plastic for bricks or pottery. Pug in Sussex meant a kind of loam.

In "Winter's Tale," iv. 2, Autolycus, referring to his molars, sings,—

> "The white sheet bleaching on the hedge,
> With, hey! the sweet birds, O how they sing!
> Doth set my pugging tooth on edge;
> For a quart of ale is a dish for a king."

Nares, in his collections, says, "There seems to be sufficient reason that it means thieving in the song of Autolycus, as pugging occurs for a thief in 'Roaring Girl.'"

Then we find "toodle," a tooth; "bridle-teeth," bicuspids, and "snaggle-teeth," irregular teeth. To have a "love-tooth" signifies having an inclination to love; and in Lyly's "Uphues and his England" we find, "Beleeve me Philantus, I am now old, yet have I in my head a love-tooth." Gat-tooth, goat-tooth (from Saxon

*gaet*), goat-toothed, is having a licoriced tooth. Chaucer makes the wife of Bath say, "Gat-toothed I was, and that became me wele." Having a goat- or licoriced tooth signified one who was wanton or lustful. In 1742 gag-tooth signified a projecting tooth. "The poets were ill advised that fained him to be a lean, gag-toothed beldame."—NASH. Gang-teeth in animals were those which protruded from the mouth. In some instances in early England "tooth" expressed keep or maintenance. Toothy was (1) peevish or crabbed, (2) having many or large teeth; and tooth-hod signified fine pasturing.

Let us glance an instant at some ideas of the people as to irregularities of the teeth.

"Teeth wide apart is a sign of good luck" is an English saying, and the French have an equivalent. The Welsh assert that a division between the two front teeth means wealth; if you can pass a sixpenny-piece through it, wealth and wisdom are promised.

In some sections of Scotland they say, "If the front teeth are wide apart it shows there exists a fondness for the opposite sex," or, as an old woman of Aberdour expressed it, "There is an indication of lichtsome character." In other sections of Scotland this space between the incisors presages that the person will be short lived.

Having, in compliance with the directions of Mrs. Glass, of cook-memory, caught our teeth, touched upon their nomenclature, and dipped into their irregularities, what means has folk-lore furnished us to keep them from being

> "By the sharp tooth of
> Cankering eld defaced?"
> —*The Schoolmistress*, SHENSTONE.

With charms which rival and outnumber the dragon's teeth sown by Cadmus. A few of these charms follow:

A Hartford lady, eighty-four years of age, gave me this charm for permanently preventing toothache: "Get down upon your knees and pick up a bone with your teeth. Arise and take as many steps forward as you hope to live years; then return to your starting-point and deposit the bone on the earth as you removed it therefrom." There is a Chinese superstition that extract of dandelion renews the youth, hair, and teeth. If you pick your teeth with the nail from the middle toe of an owl, you will never have toothache.

It is one of the superstitions of the Rio Grande that if you cut your finger-nails every Friday you will never have toothache. The same idea prevailed among the Pennsylvania Dutch. A variant given me by an old nurse directs that you must never cut the finger-nails of a child before it is a year old, for fear it will become a thief.

In the "far East" it is believed that the child will be exposed to wickedness if its nails are cut during its first year, but after that age regular cutting on Friday preserves it from toothache.

The Tuscarora Indians had a custom which they supposed would keep the teeth in their normal condition through life. A snake was held at length by its head and tail. One should bite into it all along the back-bone from head to tail, and thenceforth perfect teeth would be your possession.

I have many other notes in my collection (some of which I have published) showing this to be a quite common custom. A charm to prevent toothache in Wiltshire, England, "is a want's (*i.e.*, a mole) fore-legs and one of his hind-legs worn around the neck in a bag." The Romans used to hang beads of coral on the cradles and around the necks of infants, to "preserve and fasten their teeth" and "save them from the falling sickness." This amulet was also a preventive for various ills. In Cornwall, England, toothache was speedily and permanently cured by biting from the ground the first fern that appeared "in the spring." In "The General Dispensatory Containing the Doses, Virtues, and Uses of the Simples and Compounds, 3rd ed., London, 1773," is the following:

"*Lapis Medicamentosus* (*the Medicinal Stone*).—Take Alum, Litharge, Bole-Armenic or French Bole, Colcothar of Green Vitriol, of each, three ounces; of Vinegar, a quarter of a pint. Mix and evaporate the moisture 'till they grow hard. The use of this is to fasten the teeth, preserve the gums, to heal and dry up wounds and ulcers. It is also employed in injections and eye waters."

Should these prophylactic measures prove inefficient, in lieu of the large number of "new remedies" which are continually putting in an appearance, let me recommend some of the following, which have at least the prestige of antiquity, and will probably, in many cases, be quite as reliable as some of our modern "cures."

Among the many musty tomes into which I have dipped is the first medical work printed in the English language, and entitled

### The Breuyary of Helthe

—IMPRYNTED at LONDON—

in Fleteſtrete at the Syne
of the George next to
Saynt Dunſtones churche
by Wyllym Myddelton in the
yere of our Lorde
Mccccxlvii
the xv daye
of July

### The-97-Capytle doth ſhew of a mannes tothe

A tothe Dens is the latyn word. In greke it is named Odons. In englysh it is named a tothe. A tothe is a ſenſyble bone, the which beyng in a lyvinge mannes heed hath felynge, and so hath none other bone in mannes body, and therefore the tothe ache is an extreme payne

### ¶ The cauſe of this payne

This payne dothe come either by a humour deſcendyng out of the heed to the teeth or gumes, or it may come by corodyng, or eatyng of wormes, or it may come of corruption lyenge and beynge upon and betwyn y^e teeth, or it may come by drynkynge of hote wynes, eatynge of hoote ſpyces, or eatynge of hote aples, peares and such lyke, or it may come of a hote lyuer or ſtomake.

### ℭ A remedy

Fyrst purge the heed with pilles of cochte. And ufe gargaryces. And if it do come of any colde caufe, chew in the mouth diuers tymes the roote of horehounde. And if it do come by wormes, make a candell of waxe with henbane fedes, and lyght it, and let the perfume of the candyl enter y^e toth, and gape ouer a dyffhe of colde water & then may you take the wormes out of the water and kyl thē on your nayle, the worme is lytle greater than y^e worme in a mannes hande and beware of pullynge out any tothe, for pull out one and pul out mo. To mundyfy the teeth waffhe them eury mornynge with colde water and a lytle roche alome.

In Newfoundland "toothache is charmed away by muttering certain words while applying the finger to the spot, or by tying so many knots on a fishing-line. But the most effective cure for it is a written charm inclosed and sealed up, the contents of which must be concealed from the party afflicted, and worn around the neck." The following is a copy of one of these:

> "I've seed it written a feller was sittin'
> On marvel [marble] stone and our Lord came by;
> And He said to him, What's the matter with thee, my man?
> And he said, Got the toothache, marster.
> And He said, Follow me, and thee shall have no more toothache."

"A woman of Chance Cove, Newfoundland, said she had tried everything for this 'hell of all diseases.' She had worn 'Our Lord's letter' for a fortnight without avail."

In my collection these exhortations are in great variety, and from some such one as the following, used in Devonshire, England, they probably all originated:

"All glory! all glory! all glory! be to the Father and to the Son and to the Holy Ghost." "As our Lord and Saviour Jesus Christ was walking in the garden of Gethsemane, He saw Peter weeping. He called him unto Him and said, Peter, why weepest thou? Peter answered and said, Lord, I am grievously tormented with pain, the pain of my tooth. Our Lord answered and said, If you will believe in me, and my words abide with thee, thou shalt never feel any more pain in thy tooth. Peter said, Lord, I believe, help my unbelief, in the Name, &c.

"God grant M. N. ease from pain in the teeth."

Amber was said to have great electric and medicinal power when worn as beads around the neck and pulse. It would cure sore throat, ague, and toothache, and would drive away snakes.

Buffon, in his "Natural History," says, "The people believe that a piece of the rope with which a criminal has been hung is a cure for quartan fever, colic, sciatica, and toothache." In Spain, to kiss an unbaptized child before any one else has done so is a panacea against toothache. In South Northampton, England, "A tooth taken from the mouth of a corpse, enclosed in a bag, to be suspended from the neck, was esteemed highly." In Staffordshire it was carried in the pocket. A Newfoundland man, as a last resort to cure this "ugly monster," scraped some dust off a tombstone and drank it in water, without effecting a cure.

Among the Indians of Connecticut, according to De Forest, "toothache seems to have been common; and Roger Williams records the ludicrous fact that, while they could endure every other pain with fortitude, this was too much for their resolution, and would make them cry and groan after a most piteous fashion." For curatives they employed sweating and purgative herbs, but placed most reliance upon "a set of men called *powwows*."

The natives of the Rio Grande made use of a tea of the little lemon-perfumed berries of the "colima." A favorite early English cure was to drive a nail, sometimes taken from a coffin, into an oak-tree.

In various portions of the world a worm is considered the cause of this dire malady.

In Germany the pear-tree was appealed to:

> "Pear-tree, I complain to thee;
> Three worms sting me."

The Chinese, as you know, believe in the worm, and in New Zealand this charm was used:

> "An eel, a spineyback;
> True indeed, indeed: true in sooth, in sooth.
> You must eat the head
> Of said spineyback."

Shakespeare says,—

> "*D. Pedro.* What! sigh for the toothache?
> *Leonato.* Where is but a humour or a worm."

In Derbyshire, to extract the worm, "a small quantity of a mixture of dried and powdered herbs was placed in a teacup or other small vessel and a live coal from the fire was dropped into it. The patient then held his or her open mouth over the cup and inhaled the smoke as long as it could be borne. The cup was then taken away, and a fresh cup or glass containing water was put before the patient. Into this cup the person breathed hard for a few moments, and then it was supposed the grub or worm could be seen in the water.

In Orkney toothache is called the worm, and, as a remedy, what is known as "wormy lines" is written on paper and carried about as a charm.

Among other resources may be mentioned: Rubbing the gums with an ant, bee, lady-bug, or fly, and carrying double nuts in the pocket. The spine of a dog-fish, kissing a mule, burying a tooth in the hole of a mouse. Carrying as a talisman the tooth of a soldier killed in battle, or that of a murdered man, or pricking the gum with a sharp twig from a sweet apple-tree.

Should all these fail and you desire to be rid of the offender, try this specific from a manuscript dated 1610, and published in the *Gentleman's Magazine*, 1835:

" *To Make an Aching Tooth fall out.*

"Take wheate meal, and mix thoroughly with milk of the hearbe called spurge, and make thereof a paste of doughe, with which ye shall fill the hollowe of the tooth, and let it be there a certain time, and the tooth will fall out of itself. Also, if you wash your mouth with wine wherein the root of this hearb hath bene sodden, you will never have payne in your teethe."

To close this section of "Tothe-Lore" without a brief reference to St. Apollonia would be an act of discourtesy to her memory, as she was believed to have great sympathy for all who suffered the torments of toothache and other pains of the jaw.

Part of her martyrdom consisted in submitting to barbarous extraction of her teeth at the hands of her tormentors, and it is naturally supposable that fellow-feeling made her wondrously kind.

Her emblems are described variously to be "holding a tooth in pincers; her teeth pulled out; pincers in left hand, tooth in right; pincers alone; tied to a pillar and scourged. When Sampson Carasso bids Don Quixote's housekeeper to get him "something warm for breakfast and by the way repeat St. Apollonia's orison," the good housekeeper objects. "Dear me . . . the orison of Saint Apollonia, say you. That might do something if my master's distemper lay in his teeth, but, alas! it lies in his brain."

This is from Charles Jarvis's translation, edition of 1842, and the following note is appended: "The orison of Saint Apollonia (Santa Apollonia) was one of the *ensalmos* or magic skills to cure sickness, very popular in Cervantes' time." A Spanish writer, Don Francisco Berquizas, has gathered the words of this orison from the mouths of some old women at Esquiras. It is in short verses like a *sequidilla*, and the following is a literal translation of it:

"Apollonia was at the gate of Heaven and the Virgin Mary passed that way. 'Say, Apollonia, what are you about?' 'My lady, I neither sleep nor watch I am dying with a pain in my teeth.'

"'By the star of Venus and the setting sun. By the Most Holy Sacrament, which I bore in my womb, may no pain in your tooth, neither front nor back (*muela ni diente*) afflict you from this time hence forward.'"

In the time of Henry VII. it is said of the teeth of St. Apollonia which cured toothache that they would fill a tun. Rings with teeth supposed to be those of this Saint were often worn.

According to Lady Wilde's "Ancient Legends of Ireland," many miracles were also performed by the tooth of St. Patrick which fell from his mouth when he was teaching the alphabet to the new converts, and a shrine was afterwards made for the tooth, that was held in the greatest honor by the kings, chiefs, and people of Ireland.

*Query.*—Did St. Patrick have Riggs's disease? It is stated in *Chambers's Book of Days* that "the jaw-bone of Saint Patrick, enclosed in a curiously embossed silver case, has been for years in the possession of a family in humble circumstances near Belfast. This relic has long been used for a kind of extra-judicial trial, similar to the Saxon *Corsnet;* a test of guilt or innocence of very great antiquity; accused or suspected persons freeing themselves from the suspicion of crime by placing their right hand upon the reliquary and declaring their innocence, in a certain form of words supposed to be an asseveration of the greatest solemnity, and liable to instantaneous supernatural and frightful punishment, if falsely spoken, even by *suppressio veri* or *suggestio falsi.*

"It was supposed to assist women in labor, relieve epileptic fits, counteract the diabolical machinations of witches and fairies, and abate the baleful influences of the evil eye. It is not, however, of late years put to such uses, though it is still considered a most welcome visitor to a household where an immediate addition to the family is expected. " It was at one time said to contain five teeth, but now retains only one, three having been given to the members of the family emigrating to America, and the fourth was deposited under the altar of the Roman Catholic Chapel of Derriaghy, when rebuilt some years ago."

I have entered so fully into those divisions of my subject so far treated this evening that I cannot, as I had expected at the outset, even touch upon many others of interest, and will content myself with simply naming a few embraced in my collection.

1. The teeth of man and various lower animals employed as charms, talismans, amulets, and remedies.
2. Teeth as weapons, articles of dress, and decoration among savages.
3. Decorative deformities of teeth among various tribes.
4. Teeth in literature.
5. Curiosities concerning the teeth of celebrities.
6. Phrases, sayings, and proverbs relating to the teeth.
7. Curious customs, myths, and superstitions regarding teeth.
8. Saliva, charms, and lore.
9. The mouth, lips, jaw, and tongue in folk-lore, and other miscellaneous subjects.

And should any one inquire why more were not presented, I will answer with an Armenian saying, " The fish was asked, Have you any news from the sea? He answered, Very much, but my mouth is full of water."

The following are the chief authorities and collections consulted in the preparation of the foregoing paper:

American Notes and Queries, vol. vii., Philadelphia, 1888–91.

Bellamy, William. Century of Charades, Boston, 1895.

Bible, the Cottage (Rev. William Patton, Ed.), Hartford, 1834.

Boorde, Andrewe. The Breyuary of Helthe, London, 1547.

Brewer, E. C. Dictionary of Phrase and Fable, last edition.

Century Dictionary, vol. x., New York (Copyright 1889–95).

Cervantes, Saavendra Miguel de. Don Quixote, Philadelphia, n. d.

Chamberlain, Alexander F. Child and Childhood in Folk-Thought, New York, 1896.

Chambers, R. Book of Days, a Miscellany of Popular Antiquities, vol. ii., Edinburgh, 1863.

Cox, Marian R. An introduction to Folk-Lore, New York, 1895.

De Forest, T. W. History of the Indians of Connecticut to 1850, Hartford, 1851.

Dunglison, R. Dictionary of Medical Science, Philadelphia, 1866.

Dyer, Thomas F. T. English Folk-Lore, second edition, London, 1880.

Folk-Lore. A Quarterly Review of Myth, Tradition, Institution, and Custom, vols. i.–ix., 1890–98. *Note.*—Organ of the English Folk-Lore Society and a continuation of the Folk-Lore Journal.

Gomme, G. L. Ethnology in Folk-Lore, New York, 1892.

Gomme, G. L. Popular Superstitions (Gentleman's Magazine Library).

Gomme, G. L. Dialect Proverbs and Word-Lore (Gentleman's Magazine Library).

Halliwell, T. O. Dictionary of Archaic and Provincial Words, 5th edition, vol. ii., London, 1865.

Husenbeth, Frederick C. Emblems of Saints, Norwich, England, 1882.

Jameson, Mrs. Anna B. Sacred and Legendary Art, Boston, 1896.

Journal of American Folk-Lore, Vols. i.–xi., 1888–98.

Kunz, G. F. Folk-Lore of Precious Stones. (*In* International Congress of Anthropology, Memoirs, Ed. C. S. Wake, Chicago, 1894.)

Lyly, J. Euphues and his England, 1580 (Arber Reprint, 1869).

Notes and Queries (London), series 1–8, 96 vols., 1850–97.

Popular Science Monthly, vol. i., New York, 1872–97.

Revue des Traditions populaires, vols. i.–xiii., Paris, 1886–98.

Shakespeare, William. Dramatic Work (Ed. George Steven), Hartford, 1837.

Shenstone, William. The Schoolmistress.

Suffolk Institute of Archæology and Natural History, 2d series, vol. ii. pp. 1–206.

Thorpe, Benjamin. Northern Mythology, London, 1851.

Trevelyan, Marie. Glimpses of Welsh Life, London, n. d.

Wilde, Jane F. S., Lady. Ancient Legends, Mystic Charms, and Superstitions of Ireland, London, 1887.

Willis, Thomas. Essay of the Pathology of the Brain and Nervous Stock, London, 1681.

